# Safety, Tolerability, and Immunogenicity of aH5N1 Vaccine in Adults with and Without Underlying Immunosuppressive Conditions

**DOI:** 10.3390/vaccines13040379

**Published:** 2025-04-01

**Authors:** Peter Malfertheiner, Eve Versage, Esther Van Twuijver, Giuliano Rizzardini, Matthew Hohenboken

**Affiliations:** 1 Department of Gastroenterology, Hepatology and Infectious Diseases, Otto von Guericke University Magdeburg, 39120 Magdeburg, Germany; 2 Seqirus, Clinical Development, Waltham, MA 02451, USA; 3 Seqirus, Clinical Development, 1105 BJ Amsterdam, The Netherlands; esther.vantwuijver@seqirus.com; 4 Department of Infectious Diseases ASST, Fatebeneftatelli Sacco, 20157 Milan, Italy

**Keywords:** pandemic influenza, pandemic influenza vaccine, H5N1, MF59 adjuvant, immunosuppressive condition, older adults

## Abstract

**Background**: Pandemic influenza may cause substantial morbidity and mortality, especially in older adults and those with immunosuppressive conditions. **Methods**: In this phase 3, stratified, randomized, controlled, observer-blind, multicenter trial, we evaluated the safety, tolerability, and immunogenicity of an adjuvanted H5N1 vaccine (aH5N1) vs. active control (MF59-adjuvanted trivalent seasonal inactivated influenza vaccine [aTIV]) in 539 adults aged 18–60 and ≥61 years. Participants were further stratified into subgroups that were healthy (18–60 years, n = 91; ≥61 years, n = 89) or had prespecified immunosuppressive conditions (18–60 years, n = 180; ≥61 years, n = 179). Antibody responses were measured with microneutralization and single radial hemolysis (SRH) assays. **Results**: aH5N1 increased antibody responses in healthy persons and those with immunosuppressive conditions in both age groups, with SRH geometric mean ratios (GMRs) > 2.5 and >2.0 in participants aged 18–60 and ≥61 years, respectively, meeting former Committee for Medicinal Products for Human Use (CHMP) criteria. Responses measured with the microneutralization and SRH assays were consistent with previous studies of aH5N1. **Conclusions**: The aH5N1 vaccine had a clinically acceptable safety and tolerability profile with an AE profile comparable to that observed in previous aH5N1 studies. These findings support the viability of aH5N1 as a pre-pandemic influenza vaccine for the immunization of at-risk individuals when an antigenically matched pandemic influenza vaccine is not yet available.

## 1. Introduction

Influenza pandemics occur when a novel influenza virus strain emerges due to genetic reassortment or mutations to zoonotic influenza viruses and is able to infect humans. When such viruses transmit efficiently from person to person, they can rapidly spread beyond the initial outbreak area and cause substantial morbidity and mortality across the world [1,2,3]. In previously unexposed populations, pandemic influenza viruses pose a threat to older adults and people with immunosuppressive and other serious medical conditions—populations typically considered vulnerable to seasonal influenza—but also to younger, healthier groups [3].

The World Health Organization (WHO) closely monitors circulating zoonotic influenza viruses, including the avian H5N1 viruses, as potential causes of future pandemics. Since 2003, the H5N1 strain has caused fewer than 1000 influenza cases worldwide, but half of these infections have been fatal [4]. Thus, pandemic preparedness efforts include the development of pre-pandemic vaccines against the H5N1 virus, among others, to protect essential workers and vulnerable populations while antigenically matched vaccines are being manufactured—a process that might take at least 4–6 months [5,6,7,8]. Use of pre-pandemic vaccines at the start of a pandemic can help immunologically prime vulnerable populations, reducing mortality, and may also reduce the chance of emergence of a reassortment pandemic strain through the vaccination of populations who are at risk of infection from both avian and human viruses (e.g., veterinarians, poultry workers, operators involved in the manufacturing of vaccines with pandemic-like strains, and laboratory workers) [3,7,8].

Both older adults aged ≥65 years and adults of any age with immunosuppression are not only more vulnerable to influenza complications but also tend to have reduced immune responses to vaccinations [9,10,11]. Enhanced seasonal influenza vaccines containing an increased antigen dose or a vaccine adjuvant are recommended to boost the immune responses in older adults [12,13]. Although immunocompromised individuals usually receive the seasonal influenza vaccine recommended for their age group, evidence suggests additional doses or an adjuvant to boost the immune response may be beneficial [9,10]. Vaccine adjuvants enhance the immune response to an antigen, making vaccines more effective in both older adults and those with immunosuppression by promoting a stronger and potentially longer-lasting immunity against diseases [14,15]. They allow for the use of smaller amounts of antigen and fewer doses, which can be particularly beneficial in improving vaccine supply and accessibility [16]. Adjuvants can be used to boost the immunogenicity of pre-pandemic vaccines while an antigenically matched vaccine is unavailable. One such adjuvant, the squalene-based oil-in-water emulsion MF59 (CSL Seqirus Inc., Waltham, MA, USA), may also have an antigen-sparing effect, meaning less antigen is needed in each vaccine dose [8,17,18]. An egg-based, MF59-adjuvanted H5N1 vaccine (aH5N1 Aflunov^®^, CSL Seqirus Inc.) is approved for immunization against the H5N1 influenza A virus in individuals 6 months and older for prophylaxis before a pandemic is declared in all EU member states, Great Britain, and Singapore. aH5N1 has been evaluated for safety in 16 clinical trials collectively, including 9688 adults and 755 children who received the vaccine.

The aim of the present study was to assess the safety, tolerability, and immunogenicity of two doses of aH5N1 given 3 weeks apart to adults aged 18–60 years and those aged ≥61 years who were healthy or who had immunosuppressive conditions. As an active control, we used a licensed MF59-adjuvanted trivalent seasonal inactivated influenza vaccine (aTIV; Fluad^®^, Seqirus, Inc., Holly Springs, NC, USA).

## 2. Materials and Methods

### 2.1. Study Design

This phase 3, stratified, randomized, controlled, observer-blind, multicenter trial was designed to evaluate the safety, tolerability, and immunogenicity of two doses of aH5N1 administered three weeks apart in healthy adults and those with immunosuppressive conditions who were stratified into two age groups: 18–60 years and ≥61 years. The study design was otherwise identical to a previously published study that evaluated aH5N1 in adults with and without high-risk medical conditions [19]. This study was conducted at nine sites in Italy, four sites in Germany, and three sites in Australia, with a follow-up of 7 months. This study was designed, implemented, and reported in accordance with the Declaration of Helsinki, the International Council for Harmonisation of Technical Requirements for Pharmaceuticals for Human Use (ICH) Harmonised Tripartite Guideline for Good Clinical Practice (GCP), and applicable local regulations. All subjects provided written informed consent.

### 2.2. Study Participants

Eligible participants were ≥18 years of age with a life expectancy of at least 12 months after study entry. Participants were recruited based on health status. Healthy subjects were enrolled based on medical evaluation, medical history and the investigator’s clinical judgment. Eligible participants with immunosuppressive conditions included adults who had received renal, cardiac, liver, lung, or bone marrow transplantation > 3 months prior to enrollment; persons with hematologic malignancies or who were receiving chemotherapy for breast, colorectal, lung, or ovarian malignancies; and adults with HIV on stable antiviral therapy with CD4+ count > 200 per mm^2^ within 3 months of enrollment and HIV viral load < 200 copies per mL within 90 days of enrollment. Patients with HIV were not permitted to have had any changes in antiviral therapy (including highly active antiretroviral therapy [HAART]) during the previous 4 weeks and/or any changes in antiviral therapy anticipated through Day 43 (3 weeks after the second dose of the vaccine); in addition, immunomodulatory therapy, including cyclosporine, products containing IgG, interleukins, interferons, or systemic glucocorticoids (including inhalatory) was not permitted within 3 months before study inclusion. All persons with immunosuppressive conditions who had other underlying diseases were required to have had stable medical control of these diseases for at least 3 months prior to enrollment, as judged by the investigator. Exclusion criteria included previous receipt at any time of an H5N1 vaccine; receipt of any other (non-study) vaccine within 7 days of either the first or second dose of the study vaccine; or a progressive or severe neurologic disorder, seizure disorder, or history of Guillain–Barré syndrome (see the Supplement for the complete list of inclusion and exclusion criteria).

Within each age stratum (18–60 or ≥61 years), participants with a prespecified immunosuppressive condition were randomly assigned to aH5N1 or aTIV in a 5:1 ratio, whereas healthy subjects were randomized 2:1 to aH5N1 or aTIV. The list of randomization assignments was produced using interactive response technology (IRT).

### 2.3. Vaccine Administration

Each 0.5 mL dose of the aH5N1 vaccine contained 0.25 mL of the adjuvant MF59 and approximately 7.5 μg HA of A/turkey/Turkey/1/2005(H5N1)-like (NIBRG-23) influenza antigen. Each 0.5 mL dose of aTIV also contained 0.25 mL of MF59; the purified viral envelope glycoproteins neuraminidase (NA) and HA, including approximately 15 μg HA of each WHO-recommended antigen for the 2014 Southern Hemisphere influenza season and the 2014–2015 Northern Hemisphere influenza season: A/California/7/2009 (H1N1)pdm09-like virus, A/Texas/50/2012 (H3N2)-like virus, and B/Massachusetts/2/2012-like virus from the B/Yamagata lineage [20,21].

Study participants received two doses of the assigned study vaccine, administered as two intramuscular injections three weeks apart (on Days 1 and 22) to the deltoid muscle, preferably of the nondominant arm. Both vaccines were provided in prefilled syringes, each with an injectable volume of approximately 0.5 mL, which were administered by unblinded study staff.

### 2.4. Procedures

With the exception of vaccine administration (which was by unblinded study staff), all study-related procedures, monitoring, and safety assessments were performed by designated blinded study staff. Blood samples were collected for immunogenicity assessments during clinic visits on Days 1 and 22 (before vaccination on those days), 43, and 202. In addition to the clinic visits, the Day 1–43 treatment period included four calls in which study staff reminded patients to fill out study-specific diary cards. The follow-up period (Days 44–202) included the Day 202 clinic visit and four safety calls.

After receiving each dose of the study vaccine, all participants remained at the study site for 30 min under medical supervision for safety monitoring. For a seven-day period after each dose, study participants recorded all solicited and unsolicited adverse events (AEs), medications, and/or other vaccines given within these time periods on diary cards. During Days 8–22 and Days 29–43, only unsolicited AEs, solicited AEs that continued beyond Day 7 or 28, and medications or vaccines given within these intervals were recorded until the next clinic visit (Days 22 and 43, respectively). Study staff documented a subset of unsolicited AEs during the follow-up period (Days 44–202) by interviewing the subject and/or reviewing available medical records.

### 2.5. Endpoints

#### 2.5.1. Immunogenicity

All immunogenicity endpoints were evaluated based on responses to A/H5N1 strains by a central laboratory (hemagglutination inhibition [HI] and microneutralization [MN] assays by Southern Research Institute Inc., Birmingham, AL, USA, and single radial hemolysis [SRH] assay by Vismederi s.r.l, Siena, Italy).

The primary objective of this study was to evaluate antibody responses to aH5N1 vaccine 3 weeks after the second vaccination according to Committee for Medicinal Products for Human Use (CHMP) criteria in place at the time the trial was conducted for adults aged 18–60 and ≥61 years as measured by HI assay against the homologous strain A/turkey/Turkey/1/2005 [22,23]. Primary endpoints included the percentage of participants achieving HI seroconversion on Day 43, the geometric mean ratio (GMR) of HI titers on Day 43/Day 1, and the percentage of participants with an HI titer ≥ 1:40 on Day 43. Seroconversion was defined as an HI titer ≥ 1:40 for subjects who were seronegative at baseline (Day 1 HI titer < 1:10) or a minimum fourfold increase in HI titer for subjects who were seropositive at baseline (Day 1 HI titer ≥ 1:10). The HI assay used in this study could not be validated to the desired level of sensitivity or accuracy at dilutions below 1:40 (i.e., for most of the HI values determined). The impact of this on the primary immunogenicity objective is unclear.

Secondary endpoints included homologous A/H5N1 antibody responses evaluated with geometric mean areas (GMAs) determined by the SRH assay on Days 22 and 43; geometric mean ratios (GMRs) determined from GMAs for Day 22/Day 1 and Day 43/Day 1; and SRH seroconversion and SRH area ≥ 25 mm^2^ on Days 22 and 43. SRH seroconversion was defined as an SRH area ≥ 25 mm^2^ for subjects who were seronegative at baseline (Day 1 SRH area ≤ 3.997 mm^2^) or a significant (at least 50%) increase in SRH area for subjects who were seropositive at baseline (SRH area > 3.997 mm^2^). SRH assessments on Day 202 were analyzed as an exploratory endpoint.

Additional exploratory endpoints included geometric mean titers (GMTs) of antibodies against the homologous A/H5N1 determined by MN assay on Days 1, 22, 43, and 202 and associated GMR values. In addition, the percentages of participants with MN titers ≥ 1:10, ≥1:40, and ≥1:80 on Days 1, 22, 43, and 202 and with a ≥4-fold increase in MN titer on Days 22, 43, and 202 were evaluated. Antibody responses to the heterologous A/H5N1 strains A/Vietnam/1203/2004XPR8 and A/Anhui/01/2005XPR8 IBCDC-RG5 were tested using the same MN and SRH endpoints.

Immunogenicity was established if all three of the following CHMP criteria for each respective age group were met [22,23]: the percentage of subjects achieving HI or SRH seroconversion was >40%, the percentage achieving an HI titer ≥ 1:40 or SRH area ≥ 25 mm^2^ was >70%, and the HI and SRH Day 43/Day 1 GMR was >2.5 for participants aged 18–60 years and if the same three measures were >30%, >60%, and >2.0, respectively, for participants ≥ 61 years of age. There are no CHMP criteria for MN. All assays were validated.

#### 2.5.2. Safety

Safety was evaluated based on percentages of participants with prespecified solicited local and systemic AEs within 7 days following each vaccination; unsolicited AEs reported within 21 days after each vaccination; and serious AEs (SAEs), new-onset chronic diseases (NOCDs), medically attended AEs, adverse events of special interest (AESIs), and AEs leading to study withdrawal collected from Day 1 through 202.

Solicited local AEs included the following injection-site reactions: erythema, induration, ecchymosis, and pain. Solicited systemic AEs included loss of appetite, nausea, fatigue, generalized myalgia, generalized arthralgia, headache, shivering/chills, vomiting, diarrhea, and body temperature ≥ 38.0 °C. The use of antipyretics or analgesics was also recorded.

### 2.6. Statistical Methods

Based on safety data from previous studies, the rate of solicited AEs was assumed to be in the range of 25% to 70%, and the rate of unsolicited AEs was assumed to be approximately 20% to 25%. For a sample size of 60 evaluable subjects (pooled healthy subjects 18–60 years of age and ≥61 years of age), the probability of observing at least one AE was 0.95 when the probability of an event was 5%. Based on these factors, the total sample size was 540, including a planned sample size of 150 aH5N1 recipients and 30 aTIV recipients from each age stratum in the subgroup with immunosuppressive conditions and 60 aH5N1 and 30 aTIV recipients from each age stratum in the healthy subgroup. No adjustments were made for dropouts in the sample size assumptions.

The immunogenicity full analysis set (FAS) included all enrolled subjects who were randomized and received at least one study vaccination and provided immunogenicity data. The per-protocol set (PPS) included all FAS subjects who received the vaccine to which they were randomized at the scheduled time points, had no major protocol deviations leading to exclusion as defined prior to unblinding/analysis, and were not excluded prior to unblinding or analysis.

Data were analyzed descriptively; no formal statistical testing was planned or performed, and thus, a hierarchical testing approach was not applied. All statistical analyses for HI and MN titers and SRH areas were performed on logarithmically (base 10) transformed HI, SRH, and MN values, were assumed to follow normal distribution, and were analyzed using an analysis of covariance (ANCOVA) model that included the vaccine-group effect and the log-transformed prevaccination antibody titer or SRH area as independent variables. Analyses were conducted by vaccine group, age stratum, and health status. Adjusted GMTs, GMRs, and two-sided 95% confidence intervals (CIs) were calculated univariately and completed by providing minimum, maximum, and median titers for the different analysis groups. Safety data were summarized for all enrolled subjects.

## 3. Results

### 3.1. Disposition and Demographics of Study Participants

**Figure 1:**
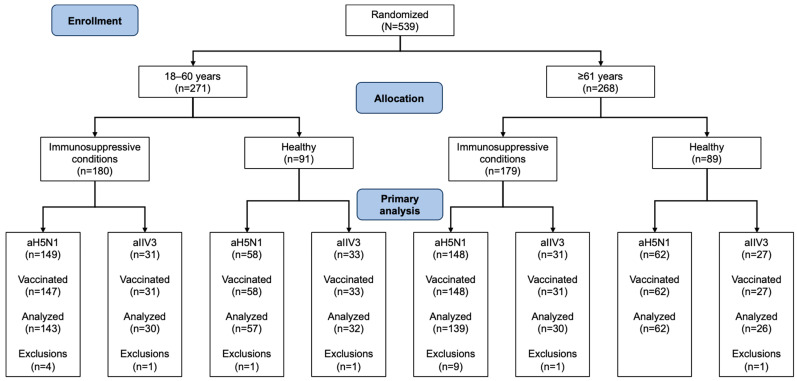
Disposition of study participants with regard to immunogenicity analyses. All exclusions were due to protocol violations.

Out of 539 study participants, 359 had immunosuppressive conditions and 180 were healthy. Age groups included 271 participants aged 18–60 years (180 with immunosuppressive conditions, 91 healthy) and 268 aged ≥61 years (179 with immunosuppressive conditions, 89 healthy). Two participants with immunosuppressive conditions in the 18–60-year age group withdrew consent prior to receiving the first dose of the study vaccine and were not included in the analysis (Figure 1). Three additional participants from this group and one from the group of healthy aTIV recipients aged 18–60 years did not receive their second vaccination.

Most participants were white and non-Hispanic, with a mean body mass index (BMI) between 25 and 29 kg/m^2^ (Table 1). Across both age groups, men comprised the majority of participants with immunosuppressive conditions. In the younger age group, those with immunosuppressive conditions tended to be older than their healthy counterparts (6- to 8-year difference in mean age), while among those aged ≥61 years, the healthy group was slightly older than the group with immunosuppression.

### 3.2. Immunogenicity Results

#### 3.2.1. Homologous Strain (A/turkey/Turkey/1/2005)

The MN and SRH assays used in this study were validated as sensitive and accurate. The HI assay used in this study (see Table S1 for results) could not be validated to the desired level of sensitivity or accuracy at dilutions below 1:40.

**Figure 2:**
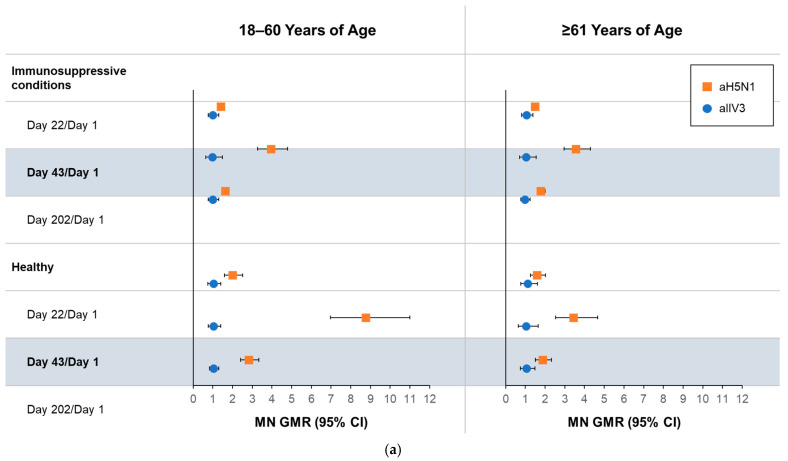

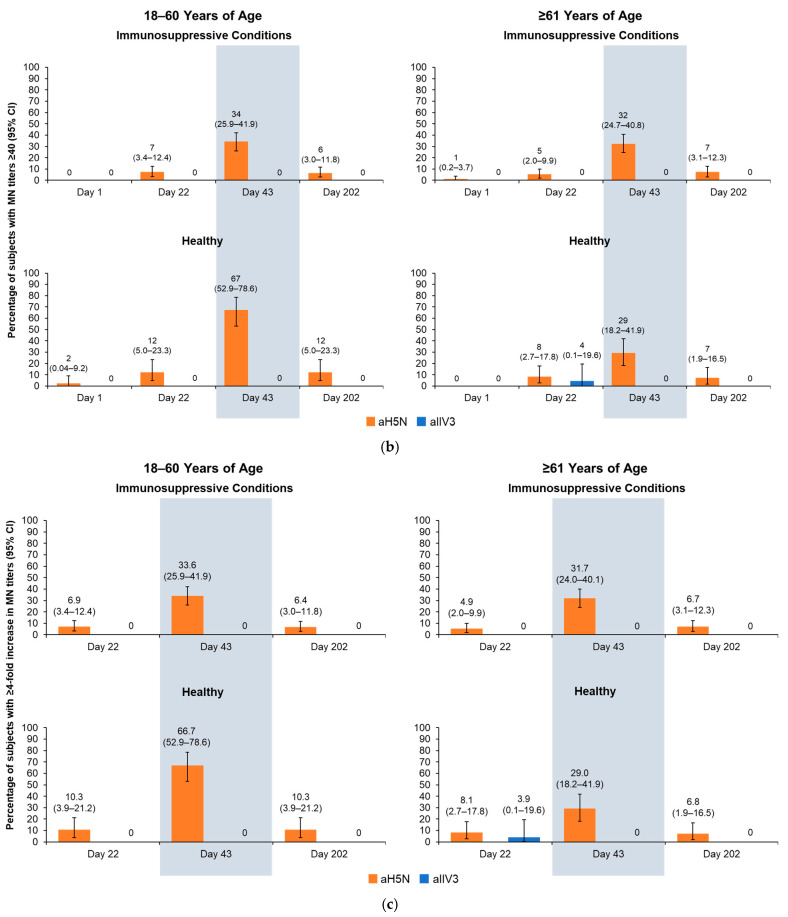
Antibody responses as measured by microneutralization (MN) on Days 1, 22, 43, and 202 in participants aged 18–60 or ≥61 years who had an immunosuppressive condition or were healthy at the time of vaccination. aH5N1 = adjuvanted H5N1 vaccine; aTIV = adjuvanted trivalent seasonal inactivated influenza vaccine: (**a**) geometric mean ratios (GMRs) of MN titers determined for Day 22/Day 1, Day 43/Day 1, and Day 202/Day 1; (**b**) percentages of subjects achieving MN titers ≥ 40 on Days 1, 22, 43, and 202; and (**c**) percentage of subjects with >4-fold increase in MN titer on Days 22, 43, and 202.

Using the MN assay (Figure 2a), recipients of aH5N1 had greater Day 43/Day 1 GMRs than aTIV recipients in all health and age subgroups. On Day 202, differences between vaccine groups remained notable, including in the group of healthy participants aged ≥61 years, with GMRs of 1.87 (95% CI, 1.50–2.33) and 1.06 (95% CI, 0.75–1.49) in the aH5N1 and aTIV subgroups, respectively (Figure 2a). None of the aTIV recipients had MN titers ≥ 40 or a ≥4-fold increase in MN titers on Day 43 or 202 (Figure 2b,c). MN titers ≥ 10, ≥20, and ≥80 in each subgroup appear in Table S2.

**Figure 3:**
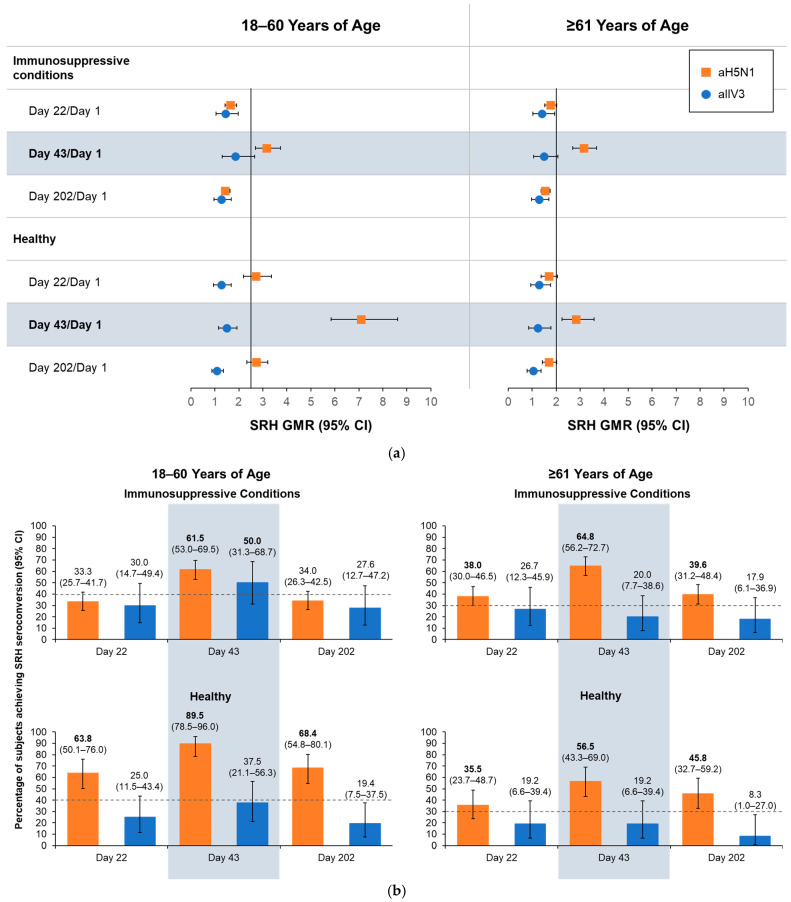

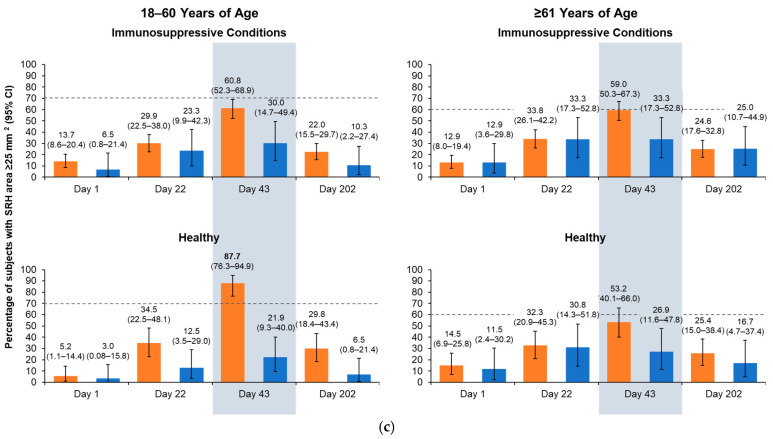
Antibody responses as measured by serial radial hemolysis (SRH) on Days 1, 22, 43, and 202 in healthy participants and those with immunosuppressive conditions who were 18–60 or ≥61 years of age at the time of vaccination. aH5N1 = adjuvanted H5N1 vaccine; aTIV = adjuvanted trivalent seasonal inactivated influenza vaccine: (**a**) geometric mean ratios (GMRs) of geometric mean areas determined for Day 22/Day 1, Day 43/Day 1, and Day 202/Day 1. Vertical lines indicate former Committee for Medicinal Products for Human Use (CHMP) criteria for each age group (18–60 years: >2.5; ≥61 years: >2.0); (**b**) percentages of subjects achieving SRH seroconversion on Days 22, 43, and 202. Dotted lines represent former CHMP criteria for each age group (18–60 years: >40%; ≥61 years: >30%); boldface indicates CHMP criteria were met; and (**c**) percentage of subjects with seroprotection (SRH area ≥ 25 mm^2^) on Days 1, 22, 43, and 202. Dotted lines represent former CHMP criteria for each age group (18–60 years: >70%; ≥61 years: >60%); boldface indicates CHMP criteria were met.

On Day 43, all aH5N1 recipients aged 18–60 years, regardless of health status, had SRH GMR values > 2.5, and those aged ≥61 years had SRH GMRs > 2, meeting former CHMP criteria (Figure 3a). At least 61.5% of aH5N1 recipients with immunosuppressive conditions from both age groups had SRH seroconversion, as did 89.5% of healthy participants aged 18–60 years and 56.5% of those aged ≥61 years (Figure 3b); thus, all age and health strata groups met the former CHMP criterion for SRH seroconversion. The proportion of healthy participants aged 18–60 years with an SRH area ≥ 25 mm^2^ was 87.7%, whereas proportions in the other subgroups were below the CHMP thresholds of 70% for 18–60 years and 60% for those aged ≥61 years (Figure 3c).

#### 3.2.2. Heterologous Strains

When tested against the heterologous strains Vietnam/2004 and Anhui/2005, Vietnam/2004, aH5N1-induced antibody responses tended to be greater in healthy participants aged 18–60 years compared with the other subgroups. Heterologous antibody responses were similar among participants with immunosuppressive conditions in both age groups and healthy participants aged ≥61 years (Table 2). Against the Anhui/2005 strain, MN responses in younger participants with immunosuppressive conditions tended to be higher than those in older participants, and healthy participants from both age strata tended to have greater responses than their counterparts with immunosuppressive conditions (Table 2). The SRH assay results were similar among the two immunosuppressed populations and older healthy adults, while the younger healthy population had more robust responses.

### 3.3. Safety Analysis

A total of 537 participants were included in safety analyses. Over the 202-day study period, 75.2% and 73.8% of participants vaccinated with aH5N1 and aTIV, respectively, had at least one solicited or unsolicited AE (excluding SAEs) that was reported by >5% of the study population, while 14 (3.4%) aH5N1 recipients and 5 (4.1%) aTIV recipients reported an SAE. One SAE (occurred after the second vaccination with aH5N1) was considered possibly related to vaccination by the investigator. Solicited AEs were reported in 75.9% of the aH5N1 group and 73.8% of the aTIV group (Table 3). Neither of the two reported deaths was considered related to the study vaccine. A 69-year-old man with HIV died from pneumonia and congestive heart failure approximately 4 months after the second vaccination with aH5N1, and a 43-year-old man with HIV committed suicide approximately 4 months after the second aH5N1 vaccination.

**Figure 4:**
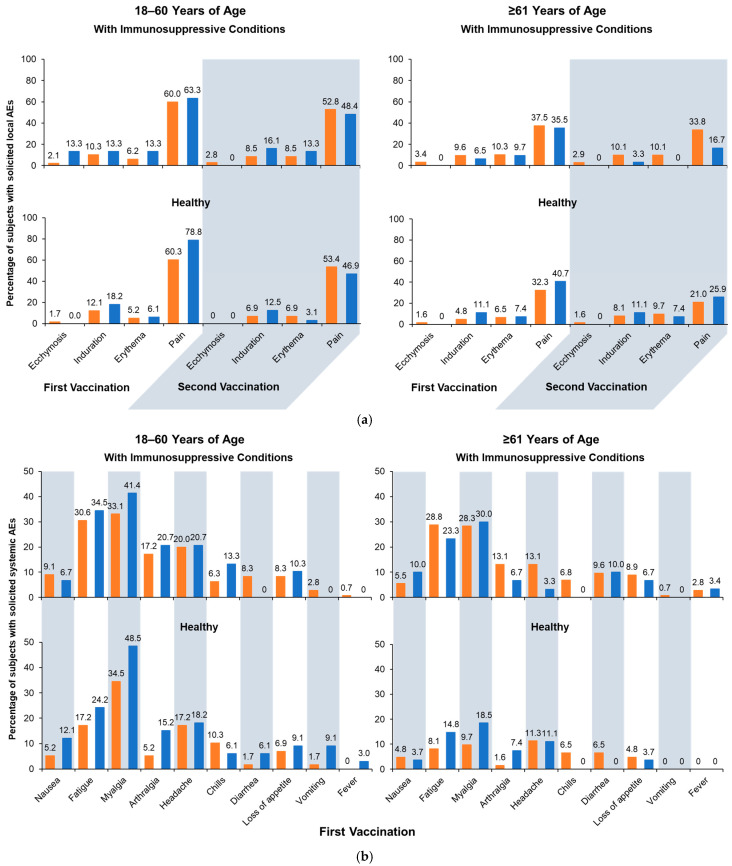

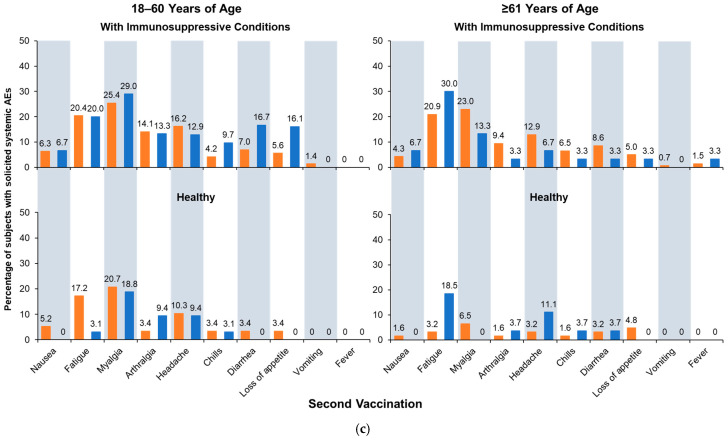
Solicited adverse events (AEs): (**a**) solicited local AEs occurring within 7 days of the first or second vaccination; (**b**) solicited systemic AEs occurring within 7 days of the first vaccination; and (**c**) solicited systemic AEs occurring within 7 days of the second vaccination. Fever is defined as body temperature ≥ 38 °C. aH5N1 = adjuvanted H5N1 vaccine; aTIV = adjuvanted trivalent seasonal inactivated influenza vaccine.

Across age and health strata, the rates of solicited AEs were generally comparable between groups receiving aH5N1 and aTIV (Figure 4). Fewer solicited local AEs were reported after the second vaccination compared with the first, and pain was the most common local AE (Figure 4a). Local AEs were mostly mild or moderate in severity, were of short duration, and occurred within 3 days of vaccination. A single severe local AE (pain) was reported after the second vaccination in a healthy subject aged 18–60 years from the aTIV subgroup.

Similar to local AE, solicited systemic AEs were generally more frequent after the first vaccination than the second, and rates were generally higher in subgroups with immunosuppressive conditions than healthy subgroups. Age differences were more pronounced in the healthy stratum, where AEs were more common among younger than older adults (Figure 4b,c) and more common in patients with immunosuppressive conditions. Across all subgroups, myalgia, fatigue, and headache were most often reported, and most events were mild or moderate, with a small number of subjects reporting severe events.

Table 4 summarizes unsolicited AEs reported between Days 1 and 43 during the trial. Among those aged 18–60 years, possibly related AEs occurred in 7.5% and 8.6% of aH5N1 recipients who had an immunosuppressive condition or were healthy, respectively, and in 9.7% and 3.0% of aTIV recipients from the same health strata, respectively. In the older age stratum, the respective rates were 11.0%, 11.3%, 9.7%, and 7.4% (Table 4). Headache was the most commonly reported possibly related unsolicited AE in the younger age stratum, and among older adults, arthralgia and pain were the most commonly reported possibly related AEs.

## 4. Discussion

In this study, antibody titers increased in people with immunosuppressive conditions as well as healthy people who were aged 18–60 and ≥61 years after a single dose and continued to rise after two doses of the pre-pandemic aH5N1 vaccine. aH5N1 was well tolerated.

The frequency of solicited and unsolicited AEs was generally comparable between groups vaccinated with aH5N1 and those receiving an adjuvanted seasonal influenza vaccine comparator, aTIV. Across age strata, AEs were more common among study participants with immunosuppressive conditions than healthy participants, and within the healthy stratum, younger participants tended to report more AEs than older adults. The frequency of AEs was comparable between younger and older age groups with immunosuppressive conditions.

Three weeks after the second vaccination with aH5N1, healthy participants aged 18–60 years met all three former CHMP SRH criteria for immunogenicity (seroconversion rate, percentage of subjects with an SRH area ≥ 25 mm^2^, and GMR) against the homologous strain A/turkey/Turkey/1/2005. Older, healthy adults (>61 yrs) and participants with immunosuppressive conditions in both age strata met two of the three former CHMP SRH criteria (seroconversion and GMR). When the MN assay was used to evaluate immunogenicity, aH5N1 elicited significantly greater Day 43/Day 1 GMRs than aTIV in all health and age subgroups, and vaccine group differences continued through Day 202. Future studies with larger sample sizes of immunocompromised individuals would be beneficial to further elucidate the immunogenicity profile in immunocompromised subpopulations.

No group met all three former CHMP HI criteria for immunogenicity three weeks after the second vaccination. Quantitative HI responses were low, which could not be validated to the desired level of sensitivity or accuracy at dilutions below 1:40 for the majority of the HI values. Influenza strains change frequently, and conducting HI assays in different laboratories remains a limitation and challenge for the field at low dilutions. Thus, the HI results were inconsistent with those observed in previous studies of aH5N1, whereas the SRH assay results were consistent with previous studies [24,25]. In addition, the SRH and MN assays used in this study were both validated as sensitive and accurate.

Heterologous antibody responses to the aH5N1 vaccine as measured by SRH and MN assays were generally lower against the Vietnam strain than the homologous Turkey strain, and heterologous antibody responses to the Anhui strain were generally lower than responses to the Vietnam strain.

## 5. Conclusions

In a population of adults with immunosuppressive conditions, aH5N1 increased antibody responses in both younger (18–60 years) and older individuals (≥61 years) as well as in healthy people in the same age groups. Responses measured with the MN and SRH assays were consistent with previous studies of aH5N1, demonstrating immunogenicity responses at levels associated with clinical protection. The aH5N1 vaccine was shown to have a clinically acceptable safety and tolerability profile with an AE profile comparable to that observed in previous aH5N1 studies.

## Figures and Tables

**Table 1:** Baseline demographics and clinical characteristics of study participants.

	18–60 Years				≥61 Years			
	Immunosuppressive Conditions		Healthy		Immunosuppressive Conditions		Healthy	
	aH5N1 (n = 149)	aTIV (n = 31)	aH5N1 (n = 58)	aTIV (n = 33)	aH5N1 (n = 148)	aTIV (n = 31)	aH5N1 (n = 62)	Ativ (n = 27)
Age, years, mean ± SD	46.2 ± 8.85	44.5 ± 8.26	37.9 ± 12.87	38.1 ± 13.31	66.2 ± 4.74	67.4 ± 5.37	69.6 ± 7.04	68.6 ± 7.02
BMI, kg/m^2^, mean ± SD	25.0 ± 4.09	25.2 ± 4.09	24.6 ± 5.31	26.2 ± 5.41	26.3 ± 4.28	26.0 ± 4.03	27.6 ± 5.51	29.3 ± 7.40
Male sex, n (%)	127 (85.2)	24 (77.4)	22 (37.9)	18 (54.5)	135 (91.2)	28 (90.3)	33 (53.2)	11 (40.7)
Race and ethnicity, n (%)								
Asian	3 (2)	1 (3.2)	0	0	1 (0.7)	0	0	0
Black	6 (4)	3 (9.7)	1 (1.7)	1 (3)	1 (0.7)	1 (3.2)	0	0
Native Pacific Islander	2 (1.3)	0	0	0	0	0	0	0
White	136 (91.3)	27 (87.1)	57 (98.3)	32 (97)	142 (95.9)	30 (96.8)	62 (100)	27 (100)
Other	2 (1.3)	0	0	0	4 (2.7)	0	0	0
Hispanic or Latino ethnicity	9 (6)	4 (12.9)	2 (3.4)	0	4 (2.7)	0	0	0
Immunosuppressive conditions, n (%)								
HIV	143 (96)	31 (100)	0	0	131 (88.5)	25 (80.6)	0	0
Transplant	1 (0.7)	0	0	0	5 (3.4)	1 (3.2)	0	0
Cancer ^a^	5 (3.4)	0	0	0	12 (8.1)	5 (16.1)	0	0

Abbreviations: aH5N1 = adjuvanted H5N1 pandemic influenza vaccine; aTIV = adjuvanted trivalent seasonal inactivated influenza vaccine; BMI = body mass index; SD = standard deviation. ^a^ Hematologic malignancy or receiving chemotherapy for breast, colorectal, or ovarian malignancies.

**Table 2:** Antibody responses to heterologous strains.

Result (95% CI)	18–60 Years		≥61 Years	
	Immunosuppressive Conditions (n = 146)	Healthy (n = 58)	Immunosuppressive Conditions (n = 147)	Healthy (n = 62)
Vietnam/2004				
MN Assay				
GMT, Day 1	5.70 (5.42–5.99)	5.60 (5.16–6.07)	6.17 (5.69–6.69)	6.43 (5.76–7.17)
GMT, Day 43	8.59 (7.74–9.54) (n = 143)	11.46 (9.67–13.58) (n = 57)	9.96 (8.76–11.31) (n = 139)	9.75 (7.99–11.88)
GMR, Day 43/Day 1	1.51 (1.36–1.68) (n = 143)	2.05 (1.73–2.43) (n = 57)	1.57 (1.38–1.79) (n = 139)	1.56 (1.28–1.91)
Percentage with MN titers ≥ 40, Day 43	5.0 (2.0–9.8) (n = 143)	11.0 (4.0–21.5) (n = 57)	6.0 (3.0–11.9) (n = 139)	6.0 (1.8–15.7)
Percentage with ≥4-fold increase in MN titers, Day 43	4.9 (2.0–9.8) (n = 143)	8.8 (2.9–19.3) (n = 57)	5.0 (2.0–10.1) (n = 139)	6.5 (1.8–15.7)
SRH Assay				
GMA, Day 1	8.03 (7.06–9.13)	6.83 (5.93–7.85)	7.93 (7.01–8.97)	8.21 (6.75–9.99)
GMA, Day 43	13.87 (11.92–16.14) (n = 143)	21.75 (17.55–26.97) (n = 57)	14.44 (12.50–16.69) (n = 139)	15.35 (12.54–18.79)
GMR, Day 43/Day 1	1.76 (1.51–2.05) (n = 143)	3.26 (2.63–4.04) (n = 57)	1.80 (1.55–2.08) (n = 139)	1.90 (1.55–2.33)
Percentage with SC, Day 43	39.9 (31.8–48.4) (n = 143)	71.9 (58.5–83) (n = 57)	38.1 (30.0–46.7) (n = 139)	43.6 (31.0–56.7)
Percentage with SRH area > 25 mm^2^, Day 43	35.0 (27.2–43.4) (n = 143)	54.4 (40.7–67.6) (n = 57)	33.8 (26–42.3) (n = 139)	32.3 (20.9–45.3)
Anhui/2005				
MN Assay				
GMT, Day 1	5.51 (5.25–5.78)	5.34 (5.00–5.70)	5.55 (5.29–5.81)	5.38 (5.06–5.71)
GMT, Day 43	8.92 (7.95–10.01) (n = 143)	12.67 (10.48–15.33) (n = 57)	8.77 (7.83–9.82) (n = 139)	9.52 (7.72–11.74)
GMR, Day 43/Day 1	1.63 (1.46–1.83) (n = 143)	2.40 (1.98–2.90) (n = 57)	1.57 (1.40–1.76) (n = 139)	1.76 (1.43–2.17)
Percentage with MN titers ≥ 40, Day 43	8.0 (3.9–13.3) (n = 143)	9.0 (2.9–19.3) (n = 57)	5.0 (2.0–10.1) (n = 139)	6.0 (1.8–15.7)
Percentage with ≥4-fold increase in MN titers, Day 43	7.0 (3.4–12.5) (n = 143)	8.8 (2.9–19.3) (n = 57)	5.0 (2–10.1) (n = 139)	6.5 (1.8–15.7)
SRH Assay				
GMA, Day 1	7.04 (6.34–7.82)	6.48 (5.75–7.31)	6.86 (6.21–7.59)	7.76 (6.47–9.31)
GMA, Day 43	10.51 (9.17–12.04) (n = 143)	15.04 (12.26–18.44) (n = 57)	11.35 (9.91–13.00) (n = 139)	10.97 (9.06–13.27)
GMR, Day 43/Day 1	1.51 (1.31–1.72) (n = 143)	2.48 (2.03–3.04) (n = 57)	1.70 (1.48–1.94) (n = 139)	1.50 (1.24–1.81)
Percentage with SC, Day 43	31.5 (24.0–39.8) (n = 143)	59.7 (45.8–72.4) (n = 57)	36.7 (28.7–45.3) (n = 139)	29.0 (18.2–41.9)
Percentage with SRH area > 25 mm^2^, Day 43	23.8 (17.1–31.6) (n = 143)	28.1 (17.0–41.5) (n = 57)	23.0 (16.3–30.9) (n = 139)	24.2 (14.2–36.7)

Abbreviations: GMA = geometric mean area; GMR = geometric mean ratio; GMT = geometric mean titer; MN = microneutralization; SC = seroconversion; SRH = single radial hemolysis.

**Table 3:** Overall summary of solicited and unsolicited AEs.

AE, n (%)		aH5N1 (n = 415)	aTIV (n = 122)
Any ^a^		312 (75.2)	90 (73.8)
Solicited AE	Any	315 (75.9)	90 (73.8)
	Local	285 (68.7)	83 (68.0)
	Systemic	222 (53.5)	65 (53.3)
	Analgesic/antipyretic use	24 (5.8)	8 (6.6)
Unsolicited AE	Any	175 (42.4)	41 (33.6)
	Severe	22 (5.3)	6 (4.9)
	Related	43 (10.4)	9 (7.4)
	Leading to study withdrawal, excluding deaths	4 (1.0) ^b^	0
SAE	Any	14 (3.4)	5 (4.1)
	Related	1 (0.2)	0
Medically attended AE		154 (37.4)	35 (28.7)
AESI		0	0
NOCD		13 (3.1)	1 (0.8)
Death		2 (0.5)	0

Abbreviations: AE = adverse event; AESI = adverse event of special interest; aTIV = adjuvanted trivalent seasonal inactivated influenza vaccine; NOCD = new-onset chronic disease; SAE = serious adverse event. ^a^ Reported by >5% of subjects, excluding SAEs. ^b^ Includes two subjects for whom an adverse event was the primary reason for study discontinuation.

**Table 4:** Unsolicited AEs occurring between Days 1 and 43 ^a^.

	All AEs, n (%)				At Least Possibly Related AEs, n (%)			
	Immunosuppressive Conditions		Healthy		Immunosuppressive Conditions		Healthy	
18–60 Years	aH5N1 (n = 147)	aTIV (n = 31)	aH5N1 (n = 58)	aTIV (n = 33)	aH5N1 (n = 147)	aTIV (n = 31)	aH5N1 (n = 58)	aTIV (n = 3)
Any	44 (29.9)	10 (32.3)	13 (22.4)	4 (12.1)	11 (7.5)	3 (9.7)	5 (8.6)	1 (3.0)
Upper respiratory tract infection	5 (3.4)	2 (6.5)	1 (1.7)	0	1 (0.7)	1 (3.2)	1 (1.7)	0
Nasopharyngitis	3 (2.0)	0	1 (1.7)	0	0	0	0	0
Back pain	3 (2.0)	1 (3.2)	0	0	0	0	0	0
Headache	2 (1.4)	1 (3.2)	3 (5.2)	0	2 (1.4)	0	1 (1.7)	0
**≥61 Years**	**aH5N1** **(n = 146)**	**aTIV** **(n = 31)**	**aH5N1** **(n = 62)**	**aTIV** **(n = 27)**	**aH5N1** **(n = 146)**	**aTIV** **(n = 31)**	**aH5N1** **(n = 62)**	**aTIV** **(n = 27)**
Any	56 (38.4)	9 (29.0)	13 (21.0)	3 (11.1)	16 (11.0)	3 (9.7)	7 (11.3)	2 (7.4)
Upper respiratory tract infection	5 (3.4)	0	2 (3.2)	1 (3.7)	0	0	1 (1.6)	1 (3.7)
Fatigue	5 (3.4)	1 (3.2)	0	0	2 (1.4)	0	0	0
Arthralgia	4 (2.7)	1 (3.2)	0	0	3 (2.1)	0	0	0
Headache	4 (2.7)	0	0	0	1 (0.7)	0	0	0
Bronchitis	3 (2.1)	0	0	1 (3.7)	1 (0.7)	0	0	1 (3.7)
Pain	3 (2.1)	0	0	0	3 (2.1)	0	0	0
Myalgia	3 (2.1)	1 (3.2)	0	0	2 (1.4)	0	0	0

Abbreviations: AE = adverse event; MedDRA = *Medical Dictionary for Regulatory Activities*. ^a^ “Any” refers to unsolicited AEs classified by MedDRA system organ class with a frequency ≥ 5% after any vaccination. Unsolicited AEs listed by MedDRA preferred term include those occurring at least twice in any group after any vaccination.

## Data Availability

The data that support the findings of this study are available from the corresponding author upon reasonable request.

## Supplementary Materials

The following supporting information can be downloaded at https://www.mdpi.com/article/10.3390/vaccines13040379/s1, Inclusion and Exclusion Criteria; Table S1: HI antibody response against homologous strain (A/turkey/Turkey/1/2005) in the full analysis set; Table S2. Subjects with MN titers ≥ 10, ≥20, and ≥80 against the homologous strain (A/turkey/Turkey/1/2005) in the full analysis set.
