# Supplementary material for: Safety, Tolerability, and Immunogenicity of aH5N1 Vaccine in Adults with and Without Underlying Immunosuppressive Conditions
Source: Vaccines (Basel). 2025 Apr 1;13(4):379. doi: 10.3390/vaccines13040379 (PMC12030940; doi:10.3390/vaccines13040379)
Supplement: Supplementary file 1 [file vaccines-13-00379-s001.zip › vaccines-3461229-supplementary.pdf]

SUPPLEMENT TO:

Safety, Tolerability, and Immunogenicity of aH5N1 in Adults With and Without Underlying Immunosuppressive Conditions

Peter Malfertheiner, Esther Van Twuijver, Eve Versage, Giuliano Rizzardini, and Matthew Hohenboken

INCLUSION AND EXCLUSION CRITERIA

Inclusion Criteria

In order to be eligible for participating in this study, a subject had to meet ALL of the following inclusion criteria in the "All Subjects" section and ALL of the inclusion criteria in the respective section for his/her health status (healthy or with immunosuppressive condition):

All Subjects

1. Male and female subjects 18 years of age and older at the time of enrollment who were noninstitutionalized, mentally competent, willing and able to understand the nature and risks of the proposed study, and able to sign the consent form prior to study entry.
2. Subjects with a projected life expectancy of 12 months or longer.
3. Subjects who were able to comply with all study procedures and requirements.

Subjects With Immunosuppressive Conditions Only

4. Subjects with a confirmed diagnosis of only one of the following medical conditions:
   a. Transplant recipients: Adult subjects who have undergone prior renal, cardiac, liver, lung, or bone marrow transplantation, more than 3 months prior to enrollment;
   b. HIV-infected subjects:
      - Adults with confirmed diagnosis of HIV infection,
      - CD4+ cell count >200 per mm3 within 3 months prior to the study enrollment,
      - HIV viral load <200 copies/mL within 90 days prior to the inclusion in the study,
      - No changes in the antiviral therapy (including highly active antiretroviral therapy (HAART) during the previous 4 weeks and/or change in the antiviral therapy anticipated through Day 43 (3 weeks after the second dose of the vaccine),
      - No use of immunomodulatory therapy, including cyclosporine, products containing IgG, interleukins, interferons, or systemic glucocorticoids (including inhalatory) within 3 months before study inclusion,
   c. Subjects with hematologic malignancies or subjects who are receiving chemotherapy for breast, colorectal, lung, or ovarian malignancies.
5. Subjects with above-mentioned immunosuppressive conditions with other underlying diseases who had stable medical control of these diseases for at least 3 months prior to enrollment in the judgment of the Investigator.

Healthy Subjects Only

6. Subjects who were in good health as determined by the outcome of medical history, physical assessment, and clinical judgment of the Investigator.

Exclusion Criteria

In order to be eligible for participating in this study, a subject should have met NONE of the following exclusion criteria in the "All Subjects" section and NONE of the exclusion criteria in the respective section for his/her health status (healthy or with immunosuppressive condition):

All Subjects

1. Subjects who were not able to follow all the required study procedures for the whole period of the study.

2. Subjects with behavioral or cognitive impairment or psychiatric disease that, in the opinion of the Investigator, could interfere with the subject's ability to participate in the study.
3. Subjects who were hospitalized or residing in a nursing care facility.
4. Subjects who were planning to change their home address due to relocation during the course of the study and could be unavailable for follow-up.
5. Subjects with any fatal prognosis of an underlying medical condition (<12 months life expectancy).
6. Subjects with any progressive or severe neurologic disorder, seizure disorder, or history of Guillain-Barré syndrome.
7. Subjects who had received other (nonstudy) vaccines within 7 days of either Day 1 or Day 22 vaccination.
8. Subjects who had ever received an H5N1 vaccine.
9. Subjects who had received another investigational product within 30 days prior to Day 1 or before completion of the safety follow-up period in another study and who were unwilling to refuse participation in another clinical study at any time during the conduct of this study.
   Note: Concomitant participation in an observational study (not involving drugs, vaccines, or medical devices) was acceptable.
10. Subjects with a history of any anaphylaxis, serious vaccine reactions, or hypersensitivity to any of the following: influenza viral proteins, excipient(s) of the study or reference vaccine, eggs (including ovalbumin), or chicken protein.
11. Subjects with a history of (or current) drug or alcohol abuse that, in the Investigator's opinion, could have interfered with the subject's safety or evaluation of the study objectives.
12. Subjects who had to undergo surgery planned during the study period that, in the Investigator's opinion, could have interfered with the study visit schedule.
13. Subjects who were a member of the research staff or had relatives who were members of the research staff (research staff being defined as individuals with direct contact with study subjects or study site personnel with access to any study document containing subject information, including receptionists, persons scheduling appointments or making screening calls, regulatory specialists, or laboratory technicians). Hospital personnel, health care professionals, and their relatives who were not involved in this clinical study were allowed for inclusion.
14. Female subjects of childbearing potential (status post onset of menarche and not meeting any of the following conditions: menopausal for at least 2 years, status post bilateral tubal ligation for at least 1 year, status post bilateral oophorectomy, or status post hysterectomy) who were sexually active and had not used for at least 2 months prior to study entry one or more of the following acceptable contraceptive methods:
    a. Hormonal contraceptive (oral, injection, transdermal patch, implant, cervical ring),
    b. Barrier (condom with or without spermicide or diaphragm with spermicide) each and every time during intercourse,
    c. Intrauterine device,
    d. Monogamous relationship with vasectomized partner (partner must have been vasectomized for at least 6 months prior to the subject's study entry),
    e. Abstinence, if not sexually active, at least 2 months before the study entry and at least 2 months after the study entry (through Day 60 of study participation).

15. Female subjects of childbearing potential (as defined above) who had a positive pregnancy test prior to study entry, who were nursing (breastfeeding), or who were sexually active and had not used or did not plan to use acceptable contraceptive measures through Day 60 of study participation.
16. Subjects with a body temperature ≥38°C (≥100.4°F) (as measured orally) within 3 days of intended study vaccination.

Subjects With Immunosuppressive Conditions Only

17. HIV-positive subjects with CD4+ cell count ≤200 cells/mm3 or viral load ≥200 copies/mL within 3 months prior to enrollment.

Healthy Subjects Only

18. Subjects with a medical history or any illness that could, in the opinion of the Investigator, pose additional risk to the subjects due to participation in the study.
19. Subjects with a known bleeding diathesis or any condition that could be associated with a prolonged bleeding time.
20. Subjects who had a malignancy (excluding nonmelanotic skin cancer) or lymphoproliferative disorder within the past 5 years.
21. Subjects with known human immunodeficiency virus (HIV) infection or HIV-related disease.
22. Subjects who were receiving cancer chemotherapy, oral or systemic corticosteroids (topical, inhaled, and intranasal corticosteroids are permitted), or other immunosuppressive agents.
23. Subjects who received or were planning to receive blood, blood products, and/or plasma derivatives or any parenteral immunoglobulin preparation within 12 weeks prior to Day 1 or during the full length of the study.
24. Subjects who had any symptoms or diagnosis of any of the immunosuppressive conditions as described in Inclusion Criterion 4.

General note: There could have been instances when subjects met all entry criteria except one that relates to transient clinical circumstances (eg, body temperature elevation or recent use of excluded medication or vaccine). Under these circumstances, a subject could be considered eligible for study enrollment if the appropriate window for delay had passed, inclusion/exclusion criteria were rechecked, and the subject was confirmed to be eligible.

Table S1. HI Antibody Response Against Homologous Strain (A/turkey/Turkey/1/2005) in the Full Analysis Set

	**18–60 years**				**≥61 years**			
	**Immunosuppressive conditions**		**Healthy**		**Immunosuppressive conditions**		**Healthy**	
	**aH5N1 (n=149)**	**aTIV (n=31)**	**aH5N1 (n=58)**	**aTIV (n=33)**	**aH5N1 (n=148)**	**aTIV (n=31)**	**aH5N1 (n=62)**	**aTIV (n=27)**
**GMT (95% CI)**								
Day 1	5.14 (4.96-5.34)	5.00 (4.62-5.41)	5.09 (4.95-5.24)	5.00 (4.81-5.19)	5.37 (5.08-5.67)	5.29 (4.69-5.97)	5.44 (5.00-5.91)	5.00 (4.40-5.68)
Day 22	6.61 (5.83-7.48) (n=144)	5.98 (4.55-7.86) (n=30)	8.21 (6.55-10.30)	5.45 (4.02-7.39) (n=32)	7.79 (6.81-8.90) (n=142)	5.49 (4.12-7.32)	9.18 (7.02-12.01)	6.05 (3.99-9.17)
Day 43	10.31 (8.68-12.25) (n=143)	5.57 (3.82-8.12) (n=30)	18.28 (13.50-24.73) (n=57)	5.50 (3.67-8.24) (n=32)	12.76 (10.50-15.50) (n=139)	7.26 (4.78-11.04) (n=30)	15.60 (11.44-21.29)	5.59 (3.46-9.05)
Day 202	5.55 (5.17-5.96)	5.10 (4.36-5.97)	5.78 (5.04-6.63)	5.57 (4.61-6.72)	6.98 (6.25-7.79)	5.39 (4.23-6.88)	8.65 (6.84-10.95)	5.51 (3.80-7.97)
**GMR (95% CI)**								
Day22/Day 1	1.29 (1.14-1.46) (n=144)	1.17 (0.89-1.53) (n=30)	1.62 (1.30-2.04)	1.08 (0.79-1.46) (n=32)	1.47 (1.28-1.68) (n=142)	1.03 (0.78-1.38)	1.73 (1.32-2.26)	1.14 (0.75-1.73)
Day 43/Day 1	2.01 (1.69-2.39) (n=143)	1.09 (0.75-1.59) (n=30)	3.61 (2.67-4.89) (n=57)	1.09 (0.73-1.63) (n=32)	2.40 (1.98-2.92) (n=139)	1.37 (0.90-2.08) (n=30)	2.94 (2.16-4.01)	1.05 (0.65-1.71)
Day 202/Day 1	1.08 (1.01-1.16)	1.00 (0.85-1.17)	1.14 (1.00-1.31)	1.10 (0.91-1.33)	1.31 (1.17-1.46)	1.01 (0.79-1.29)	1.65 (1.31-2.09)	1.05 (0.73-1.52)
**Percentage with SC (95% CI)**								
Day 22	4.86 (2-9.8) (n=144)	3.33 (0.08-17.2) (n=30)	12.07 (5-23.3)	3.13 (0.08-16.2) (n=32)	10.56 (6-16.8) (n=142)	0	12.90 (5.7-23.9)	3.85 (0.1-19.6)

	18–60 years				≥61 years			
	Immunosuppressive conditions		Healthy		Immunosuppressive conditions		Healthy	
	aH5N1 (n=149)	aTIV (n=31)	aH5N1 (n=58)	aTIV (n=33)	aH5N1 (n=148)	aTIV (n=31)	aH5N1 (n=62)	aTIV (n=27)
Day 43	19.18 (13.1-26.5)	0	43.10 (30.2-56.8)	3.03 (0.08-15.8)	24.49 (17.8-32.3)	3.23 (0.08-16.7)	30.65 (19.6-43.7)	3.85 (0.1-19.6)
Day 202	1.42 (0.17-5)	0	3.45 (0.42-11.9)	0	5.19 (2.1-10.4)	0	16.95 (8.4-29)	0
**Percentage with HI Titer ≥1:40 (95% CI)**								
Day 1	1 (0.02-3.8)	0	0	0	1 (0.02-3.7)	0	2 (0.04-8.7)	0
Day 22	6 (2.4-10.7) (n=144)	3 (0.08-17.2) (n=30)	12 (5-23.3)	3 (0.08-16.2) (n=32)	11 (6-16.8) (n=142)	0	15 (6.9-25.8)	4 (0.1-19.6)
Day 43	20 (14-27.8) (n=143)	0 (n=30)	46 (32.4-59.3) (n=57)	3 (0.08-16.2) (n=32)	27 (20.1-35.5) (n=139)	7 (0.8-22.1) (n=30)	32 (20.9-45.3)	4 (0.1-19.6)
Day 202	2 (0.44-6.1)	0	3 (0.42-11.9)	0	7 (3.6-13.2)	4 (0.09-18.3)	17 (8.4-29)	0

Abbreviations: aH5N1 = adjuvanted H5N1 pandemic influenza vaccine; aTIV = adjuvanted inactivated seasonal influenza vaccine; CI = confidence interval; GMR = geometric mean ratio; GMT = geometric mean titer; HI = hemagglutination inhibition; SC = seroconversion, defined as an HI titer ≥1:40 for subjects who were seronegative at baseline (Day 1 HI titer <1:10) or a minimum 4-fold increase in HI titer for subjects who were seropositive at baseline (Day 1 HI titer ≥1:10).

Table S2. Subjects with MN Titers ≥10, ≥20, and ≥80 Against the Homologous Strain (A/turkey/Turkey/1/2005) in the Full Analysis Set

	**18–60 years**				**≥61 years**			
	**Immunosuppressive conditions**		**Healthy**		**Immunosuppressive conditions**		**Healthy**	
	**aH5N1 (n=149)**	**aTIV (n=31)**	**aH5N1 (n=58)**	**aTIV (n=33)**	**aH5N1 (n=148)**	**aTIV (n=31)**	**aH5N1 (n=62)**	**aTIV (n=27)**
**MN titer ≥10, number (%) of subjects [95% CI]**								
Day 1	2 (1) [0.17-4.9]	2 (6) [0.8-21.4]	3 (5) [1.1-14.4]	0	2 (1) [0.17-4.8]	1 (3) [0.08-16.7]	1 (2) [0.04-8.7]	1 (4) [0.1-19.6]
Day 22	33 (23) [16.3-30.7] (n=144)	3 (10) [2.1-26.5] (n=30)	27 (47) [33.3-60.1]	0 (n=32)	44 (31) [23.5-39.3] (n=142)	3 (10) [2-25.8]	16 (26) [15.5-38.5]	2 (8) [0.9-25.1]
Day 43	101 (71) [62.4-77.9] (n=143)	1 (3) [0.08-17.2] (n=30)	53 (93) [83.3-98.1] (n=57)	1 (3) [0.08-16.2] (n=32)	97 (70) 61.4-77.3 (n=139)	2 (7) [0.8-22.1] (n=30)	42 (68) [54.7-79.1]	1 (4) [0.1-19.6]
Day 202	55 (39) [30.9-47.6] (n=141)	1 (3) [0.09-17.8] (n=29)	49 (84) [72.6-92.7]	1 (3) [0.08-16.7] (n=31)	72 (53) [44.6-62] (n=135)	0 (n=28)	31 (53) [39.1-65.7] (n=59)	2 (8) [1-27] (n=24)
**MN titer ≥20**								
Day 1	0	0	1 (2) [0.04-9.2]	0	1 (1) [0.02-3.7]	0	0	0
Day 22	14 (10) [5.4-15.8] (n=144)	0 (n=30)	16 (28) [16.7-40.9]	0 (n=32)	16 (11) [6.6-17.7] (n=142)	0	10 (16) [8-27.7]	2 (8) [0.9-25.1]
Day 43	75 (52) [43.9-60.9] (n=143)	0 (n=30)	48 (84) [72.1-92.5] (n=57)	0 (n=32)	64 (46) [37.6-54.7] (n=139)	1 (3) [0.08-17.2] (n=30)	25 (40) [28.1-53.6]	1 (4) [0.1-19.6]
Day 202	23 (16) [10.6-23.5] (n=141)	1 (3) [0.09-17.8] (n=29)	27 (47) [33.3-60.1]	0 (n=31)	26 (19) [13-26.6] (n=135)	0 (n=28)	8 (14) [6-25] (n=59)	1 (4) [0.11-21.1] (n=24)

	18–60 years				≥61 years			
	Immunosuppressive conditions		Healthy		Immunosuppressive conditions		Healthy	
	aH5N1 (n=149)	aTIV (n=31)	aH5N1 (n=58)	aTIV (n=33)	aH5N1 (n=148)	aTIV (n=31)	aH5N1 (n=62)	aTIV (n=27)
MN titer ≥80								
Day 1	0	0	0	0	0	0	0	0
Day 22	6 (4) [1.5-8.8] (n=144)	0 (n=30)	5 (9) [2.9-19]	0 (n=32)	6 (4) [1.6-9] (n=142)	0	3 (5) [1-13.5]	0
Day 43	29 (20) [14-27.8] (n=143)	0 (n=30)	26 (47) [32.4-59.3] (n=57)	0 (n=32)	27 (19) [13.2-27] (n=139)	0 (n=30)	9 (15) [6.9-25.8]	0
Day 202	4 (3) [0.8-7.1] (n=141)	0 (n=29)	3 (5) [1.1-14.4]	0 (n=31)	3 (2) [0.46-6.4] (n=135)	0 (n=28)	3 (5) [1.1-14.1] (n=59)	0 (n=24)

Abbreviations: aH5N1 = adjuvanted H5N1 pandemic influenza vaccine; aTIV = adjuvanted inactivated seasonal influenza vaccine; CI = confidence interval; MN = microneutralization.
